# Comparison of Different Materials in the Same-Sized Cemented Stems on Periprosthetic Fractures in Bone Models

**DOI:** 10.3390/jcm14082724

**Published:** 2025-04-15

**Authors:** Kohei Hashimoto, Yukio Nakamura, Nobunori Takahashi, Takkan Morishima

**Affiliations:** 1 Department of Orthopedic Surgery, Aichi Medical University, 1-1 Yazakokarimata, Nagakute 480-1195, Aichi, Japan; skikohei4145@gmail.com (K.H.); ntakahashi0617@aichi-med-u.ac.jp (N.T.); 2 Department of Orthopedic Surgery, Division of Osteoporosis, Locomotive Syndrome, Joint Disease Center, Aichi Medical University, 1-1 Yazakokarimata, Nagakute 480-1195, Aichi, Japan

**Keywords:** bone model, cemented stem, comparison, materials, periprosthetic fractures

## Abstract

**Objective**: The increasing number of aging patients with total hip arthroplasties (THA) causes an increased incidence of periprosthetic fractures (PPF). The study aimed to evaluate the impacts of two different materials in the same-sized cemented stems on PPF in bone models. **Methods**: This study compared the maximum rotational torque leading to PPF when stems made of cobalt–chromium–molybdenum (Co–Cr–Mo) alloy and stainless use steel (SUS) were implanted using simulated bone models (Sawbones, 3403). The maximum destruction torque was compared statistically for each material (Co–Cr–Mo alloy vs. SUS stainless steel) in this model, and fracture patterns were examined. **Results**: The PPF occurred with a spiral propagation from the proximal femur towards the diaphysis, with breakage occurring near the distal end of the stem. There were no significant differences in the destruction torque values between the Co–Cr–Mo alloy (103.0 ± 14.9 Nm) and SUS (98.7 ± 15.1 Nm) samples (*p* = 0.575). The fractures using the bone models exhibited similar patterns in all specimens, resembling clinical PPF fracture types clinically, specifically Vancouver classification B2. **Conclusions**: The comparison of the maximum destruction torques of the Co–Cr–Mo alloy and SUS cemented stems in simulating PPF showed no significant differences. The results suggest that the materials of the cemented stems might not significantly affect the occurrence of PPF in THA.

## 1. Introduction 

Since the population in developed countries is aging fast and the number of THAs is increasing rapidly, it is urgently necessary to prevent various complications, such as implant migration or periprosthetic fractures (PPF), with total hip arthroplasties (THA). Current Japanese guidelines give cemented fixation a Grade B recommendation, stating that cement fixation is suggested for cases with poor bone quality or stem fit issues [1]. Fujita et al. reported favorable outcomes in their multicenter study with Exeter stems, demonstrating a 100% survival rate of the cement–bone interface at 5 years and a 98.8% reoperation-free rate. As the cohort likely included patients with osteoporosis, these favorable outcomes further support the effectiveness of cemented fixation [2]. Consequently, cemented techniques could be optimal for osteoporotic patients who need THA.

The aging population and the increasing number of patients with THA have led to an increased incidence of PPF in THA. PPFs represent a significant challenge in modern Orthopedic surgery, being a major source of patient morbidity and mortality, while simultaneously imposing substantial financial burdens on healthcare systems worldwide. Understanding the mechanism by which PPF occurs in THA has therefore become increasingly crucial for improving patient outcomes and healthcare efficiency. Recent epidemiological studies have revealed striking differences in PPF incidence between cemented and uncemented THAs. A comprehensive analysis demonstrated a Seven-fold increase in PPF incidence in uncemented THA compared to cemented THA [3], despite the growing global trend toward uncemented procedures [4]. This disparity has sparked considerable interest in understanding the underlying mechanisms of PPF formation in different prosthetic designs. Further investigations have highlighted variations in PPF risk among different stem designs. Studies indicate that cemented taper-slip stems demonstrate higher PPF risks compared to cemented composite-beam stems, with significant variations observed even among different taper-slip stem designs [5,6,7].

Multiple factors potentially contributing to PPF development have been identified through ongoing research. Stem size has emerged as one significant variable [8], while the surface characteristics at the stem–cement interface represent another crucial factor, as previously documented [9]. Of particular interest are polished tapered stems manufactured from cobalt–chromium–molybdenum (Co–Cr–Mo) alloy, which have been associated with elevated PPF risk in clinical observations [10]. Registry data reported from the United Kingdom indicate that collarless polished tapered (CPT) stems made from Co–Cr–Mo alloy have a higher likelihood of revision due to PPF compared to the Exeter stem, which is manufactured from stainless use steel (SUS) [5]. Other research groups have extensively examined the relationship between the surface roughness of metallic stem materials and bone cement. These investigations reveal that metal surface wettability decreases proportionally with increased polishing, with Co–Cr–Mo demonstrating both the lowest surface wettability and lowest frictional coefficient when compared to SUS. The authors also suggest that Co–Cr–Mo stems tend to be more slippery, resulting in greater subsidence. This suggests that the increased subsidence may contribute to a higher likelihood of fracture, indicating an elevated risk of periprosthetic femoral fracture (PPF), even though slight subsidence has been permitted as a result of creep regarding implant migration in cemented fixation based on the taper-slip concept design [11]. Despite these advances in understanding, there remains a significant gap in the literature regarding direct comparisons of different stem materials under controlled conditions. While several studies including Hirata et al. [9] and Kaneuji et al. [11] have investigated material characteristics, such as surface roughness, wettability, frictional behavior, and stem subsidence, to our knowledge, no previous studies have directly compared Co–Cr–Mo and SUS under standardized fracture testing conditions using cemented stems of identical shape and size. Our study is the first to address this gap through systematic mechanical testing. This lack of comparative data represents a crucial limitation in understanding of how material properties influence PPF risk.

This study was designed with three primary objectives. First, we conducted a comprehensive biomechanical analysis comparing the rotational stability of Co–Cr–Mo and SUS stems under identical loading conditions. Second, we evaluated the influence of surface finish characteristics on the stem–cement interface stability for both materials. We finally developed a standardized testing protocol that could be used for future comparative analyses of different stem materials and designs. Our experimental findings revealed no significant differences between these materials, suggesting that stem material composition may not significantly influence PPF incidence in THA. These results have important implications for both clinical practice and future prosthetic design considerations.

## 2. Methods

**Figure 1:**
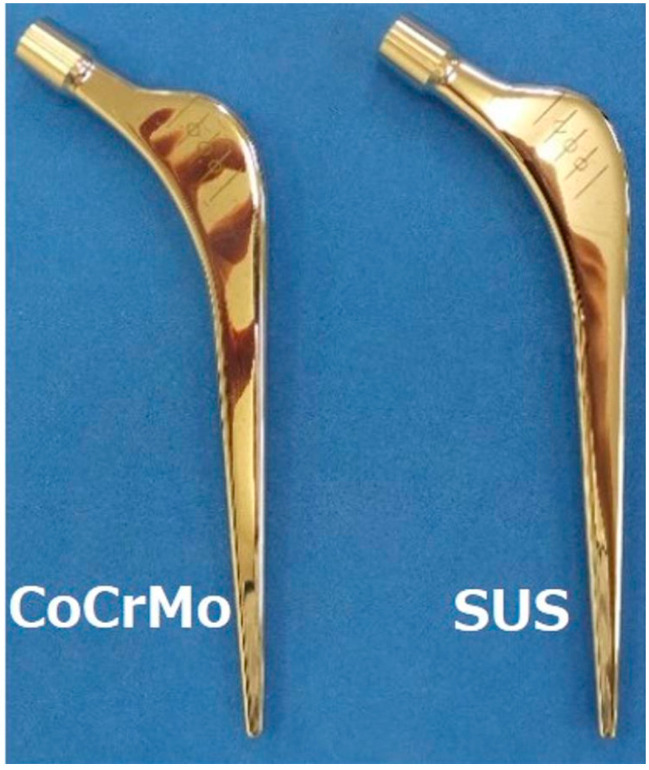
A stem made of cobalt–chromium–molybdenum (Co–Cr–Mo) alloy and a stem made of stainless use steel (SUS).

The VLIAN stem (Teijin Nakashima Medical, Okayama, Japan) is characterized by its distinctive round shoulder design specifically incorporating a gentle curved surface in the proximal lateral portion. This stem preserves surrounding bone tissue and soft tissue during surgery, and enhances stem insertability during the procedure. Furthermore, the stem features a highly polished surface treatment and employs a straight double-taper design for its body, and it is manufactured from Co–Cr–Mo alloy, providing excellent biocompatibility and durability. Through these design principles, this stem permits both minimally invasive surgery and long-term stability [12]. We first manufactured two major types of VLIAN stems made of Co–Cr–Mo alloy (ASTM F799; Teijin Nakashima Medical, Okayama, Japan) and SUS (ASTM F1586; Teijin Nakashima Medical, Okayama, Japan), respectively, both of which were the same 12/14 taper-polished stems with an offset of 45#3 (Figure 1). Both stem sizes were the same throughout the study. Note that Co–C–Mo is harder than SUS, offering the advantage of easier metal processing when manufacturing smaller-sized stems, and it also exhibits higher fatigue strength [13]. Since implant size and design likely affect outcomes, which potentially masks the influence of material properties, we investigated the effects of different material compositions of stems on fracture by measuring the destructive torque in bone models.

We then fabricated custom femoral bone models by cementing each stem into Sawbone models (Sawbones medium left femur osteoporosis model 3403; Pacific Research Laboratories, WA, USA), with eight samples, which had been assessed by power analysis, for each stem type. Based on Morishima’s paper [14], we determined that using six or more specimens would provide sufficient statistical power.

We finally compared the maximum rotational torque leading to PPF when stems made of Co–Cr–Mo alloy and SUS were implanted using those simulated bone models. The femoral models prepared using a >1 mm oversized rasp were confirmed by plain radiographs to have a consistent cement mantle surrounding the entire circumference of the stem. Also, the largest and most insertable stem was selected.

To suppress the variability of the bone resection and the initial rasping, the #45-1 specimen was developed with a machining tool (D500, Makino Milling Machine Co., Ltd., Tokyo, Japan). In addition to rasping up to the specified size 45#2~3, a cement plug was set up using an inserter (TEIJIN NAKASHIMA MEDICAL, Okayama, Japan; the diameter 13 mm, #BP190113), and the cement stem was placed using a centralizer. The procedure was performed by an experienced Orthopedic surgeon (T.M.). To ensure the proper installation of the stem, it was inserted along the insertion marking of the stem on the neck bone cutting surface of the simulated bone. The reproducibility of the methodology and fracture classification were also assessed by T.M., ensuring accurate placement and fixation using the bone cement (Surgical Simplex, Stryker, Mahwah, NJ, USA). Simplex is a medium-viscosity cement, andconsists of a polymer powder composed of 30 gram (g) of methyl methacrylate–styrene copolymer, 6 g of polymethylmethacrylate (PMMA), and 4 g of barium sulfate, mixed with a monomer liquid containing 19.5 milliliter (mL) of MMA, 0.5 mL of Newton (N), N-dimethyl-p-toluidine, and 1.5 milligram (mg) of hydroquinone. We mixed the monomer powder and polymer liquid under vacuum conditions for use. The cement hardening time was approximately 10 minutes (min) at room temperature of 21 °C. During the preparation of synthetic bone models, broaching was performed at a predetermined angle to ensure all models would be positioned similarly. The stems were inserted to the same depth using the markers designed on the stem as reference points. We then confirmed the insertion positions of the stems in the synthetic bone models using plain radiographs to verify their similar positioning. The tapered polished collarless stem used in this study was classified as a force-closed design, in which stability is maintained by a balance of forces across the stem–cement interface without a direct bond between them [15]. To evaluate the long-term stability and durability of the stem–cement interface in a clinical setting, a cement–stem debonding condition was recreated. The stem removal process was performed after confirming complete cement fixation via plain radiograph examination. Implant removal was conducted using the testing machine ElectroPuls E10000 (Instron Corp., Teijin Nakashima Medical, Okayama, Japan), which was previously used to generate the fracture by exercising a uniaxial compression force at a constant speed of 1 millimeter (mm)/min, ensuring that neither the cement mantle nor the bone sustained damage. For the test setup, we used an Instron ElectroPuls E10000 dynamic testing machine. This machine is equipped with a Dynacell load cell with a measurement range of ±10 kN for static conditions and ±7 kN for dynamic/fatigue testing. The load cell provides high-precision measurements with an accuracy of ±0.5 percent(%) of the indicated load or ±0.005% of the load cell capacity, whichever is greater. The Dynacell is a digital electronic load cell that operates with the testing system’s maximum frequency capability of 100 Helz (Hz). The load cell is PC-controlled and does not require hydraulic or pneumatic sources, operating on single-phase power. This advanced digital load measurement system is specifically designed for both static and dynamic material testing applications.

**Figure 2:**
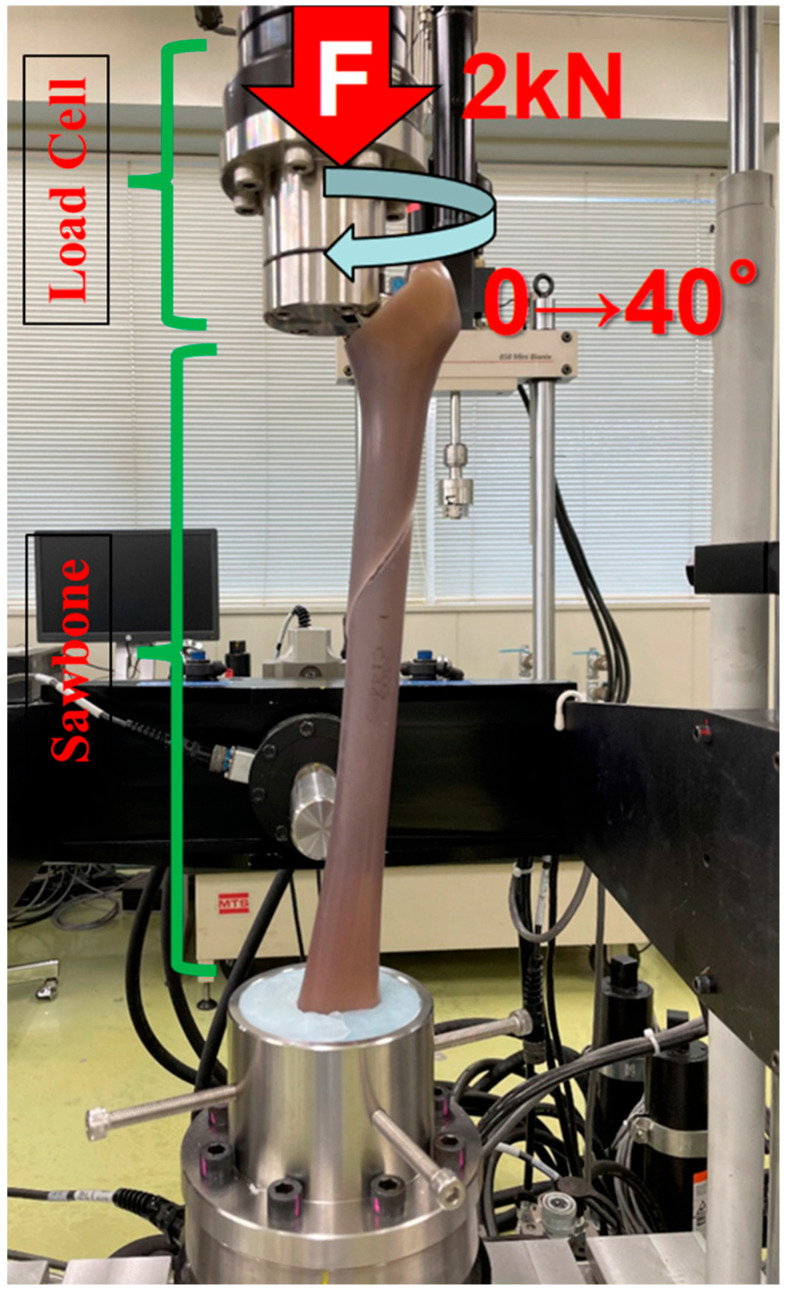
The proximal part of the simulated bone was arranged, so that the center of the cemented stem’s head was positioned along the rotational axis of the machine (Mini Bionix, MTS Systems, Eden Prairie, MN, USA). The distal end of the simulated bone was also positioned along the rotational axis and fixed with dental cement (Ostron II/GC Corporation). The rotational test combined compressive force with rotation, with an initial preload of 2 kN applied to simulate the load during a one-legged stance. While maintaining the compressive load, the cemented stem was internally rotated by 40 degrees in one second, causing a fracture in the simulated bone.

One week after stem removal, edible oil was applied to the removed stem and the stem–cement interface to simulate the moisture of intracanal fluid within the cement layer, thereby replicating a clinical environment. The stem was then reinserted and carefully aligned with the insertion marking on the cross-sectional surface of the simulated femoral neck. For the rotational test to reproduce PPF, the proximal part of the simulated bone was arranged, so that the center of the cemented stem’s head was positioned along the rotational axis of the machine (Mini Bionix; MTS Systems Corp., Eden Prairie, MN, USA). The distal end of the simulated bone was also positioned along the rotational axis and fixed with dental cement (Ostron II/GC Corporation, Alsip, IL, USA). The rotational test combined compressive force with rotation, with an initial preload of 2 kN applied to simulate the load applied when in a one-legged stance. Afterward, while maintaining the compressive load, the cemented stem was internally and constantly rotated by 40 degrees in one second, causing a fracture in the simulated bone (Figure 2). The maximum torque measured during the test was defined as the failure torque. We recorded the fracture pattern in reference to the Vancouver classification [15]. The entire methodology was set up with the standardized norms referring to Morishima et al. [16]. The data obtained from this study are expressed as mean ± standard deviation, and statistical analysis was performed using the Tukey test with a significance level of 0.05.

## 3. Results

**Figure 3:**
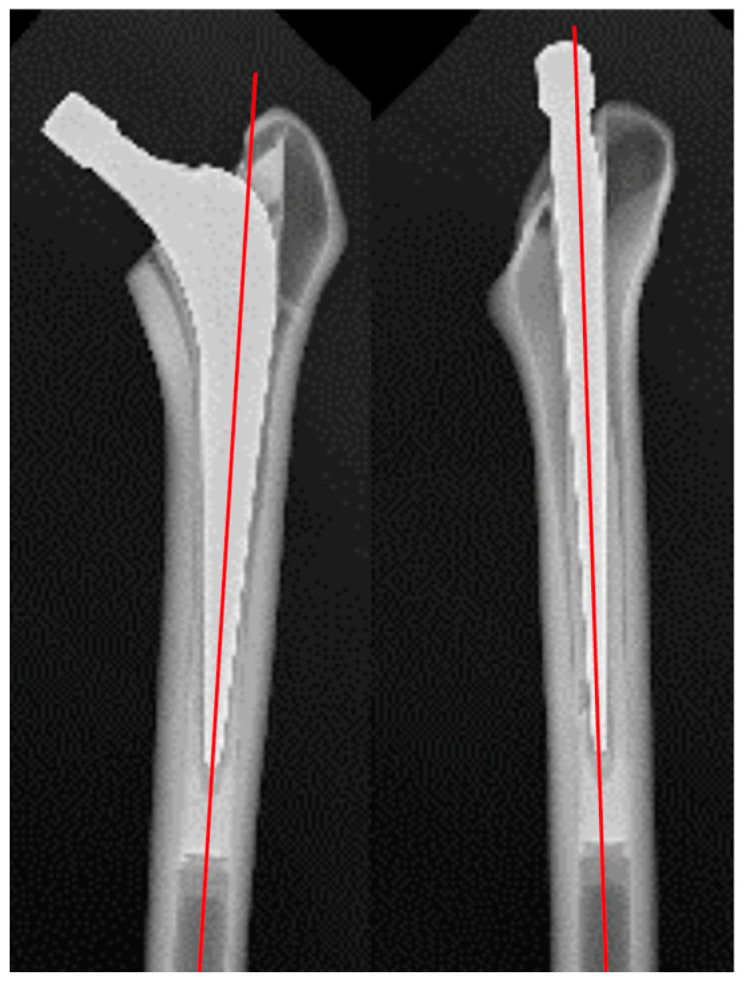
Representative anteroposterior and lateral plain radiographs showing that the alignment was within ±1 degree of the intended position.

All stems were inserted with 20 degrees of anteversion and neutral varus/valgus alignment, using the central axis of the distal tapered portion of the stem as the reference. Anteroposterior and lateral plain radiographs were examined to confirm that the alignment was within ±1 degree of the intended position (Figure 3).

**Figure 4:**
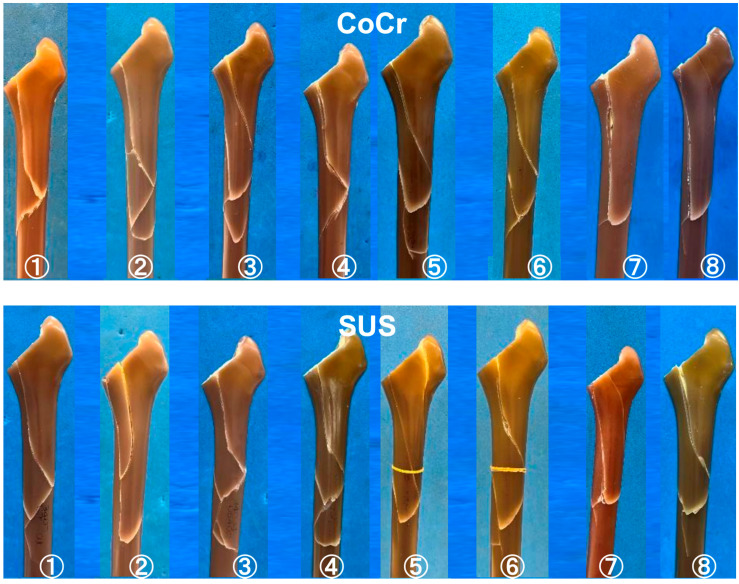
All of the fracture patterns in all specimen created by our testing mechanism.

The fracture patterns of the simulated bones propagated in a spiral manner from the posterior surface of the proximal femur to the shaft, resulting in fractures near the distal end of the stems. In all eight specimen, the direction of propagation was consistent, proceeding from proximal to distal, although there were slight differences in the lengths of the fractures (Figure 4). The fracture line began proximally and progressed to complete fracture within 2 cm of the stem tip.

A similar fracture pattern was observed in all specimens, and no differences were found based on the materials of the cemented stems.

Table 1 shows the comparative data of the failure torque during the rotational test that simulated PPF using Co–Cr–Mo alloy and SUS cemented stems. The fracture failure torque values were similar when comparing the materials of the stems (Co–Cr–Mo alloy or SUS, 103.0 ± 14.9 N·m or 98.7 ± 15.1 N·m, respectively), with no significant difference observed (*p* = 0.575). All samples were of Vancouver type B2. In the KS test of Co–Cr–Mo or SUS, the KS statistic was 0.1618 or 0.190, and the critical value was 0.457 or 0.457 for 8 samples, respectively. Since both KS statistics were smaller than the critical values, the data followed a normal distribution. Also, in the two-sample test, the KS statistic was 0.375 and the critical value was 0.678 for the eight samples. Since the KS statistic was smaller than the critical value, there was a high probability that both groups shared the same distribution characteristics.

All stem placements in the Sawbone models were satisfactory. The femoral models prepared using a >1 mm oversized rasp were confirmed by plain radiograph to have consistent cement mantle surrounding the entire circumference of each stem. After stem placement, plain radiograph examination confirmed that the cementing was successfully performed.

## 4. Discussion

In this study, we found no significant differences between Co–Cr–Mo alloy and SUS cemented stems in terms of mechanical behavior and failure characteristics. Furthermore, all specimens exhibited identical fracture patterns classified as Vancouver type B2, a consistency that provides valuable insight into the mechanical nature of these failures. These findings suggest that stem material differences may not significantly impact PPF in THA with cemented stems, challenging some prevailing assumptions in the field.

As widely reported in the literature, PPF represents a serious complication associated with significant morbidity and mortality following THA and total knee arthroplasty [17]. The clinical significance of PPF extends beyond immediate surgical complications, affecting long-term patient outcomes and healthcare resource utilization. Therefore, understanding PPF mechanisms is crucial for reducing incidence rates and improving treatment outcomes, potentially leading to enhanced QOL and life expectancy for arthroplasty patients. However, the mechanisms underlying PPF remain largely unclear, presenting a significant challenge for clinicians and researchers alike. The past decade has seen an emergence of numerous clinical and basic research reports regarding PPF [6,8,18,19], each contributing to our understanding of this complex complication. Takegami et al. provided valuable insights by reporting that differences in surface strain patterns of cemented stems might be associated with different PPF patterns [18], suggesting a mechanical basis for these failures. Collectively, it is postulated that the lower the destructive torque, the lower the resistance to PPF [12,14]. Haider et al. conducted detailed investigations showing that while cementless stems demonstrate superior biological fixation, cemented stems exhibit lower PPF risk [19], highlighting the importance of the fixation method in PPF prevention. Interestingly, Hirata et al. reported in 2021 that metal surface wettability decreases with increased polishing, with Co–Cr–Mo showing the lowest surface wettability and friction coefficient compared to SUS. This finding has particular relevance when using medium-viscosity bone cement, as Co–Cr–Mo’s low friction coefficient may induce excessive taper-slip due to reduced adhesion [9], potentially creating conditions conducive to PPF development. Kaneuji et al. conducted a biomechanical study using identical Exeter stem designs and provided important findings. They reported that the Exeter stems, which are made of Co–Cr–Mo alloy, showed significantly greater subsidence compared to SUS stems, generating more than triple the compressive force at equivalent subsidence levels. Their work also demonstrated Exeter stems’ greater propensity for sliding on cement surfaces, suggesting these characteristics might contribute to increased PPF risk [11].

Note that our study found no significant material-related differences, a noteworthy discrepancy that warrants careful consideration. Also, our results suggest that when optimal-sized stems are placed in the femur, the stem material may not significantly impact PPF, challenging existing paradigms in implant design and material selection. However, this outcome may be size-dependent, with potentially different results for smaller sizes, highlighting the need for the careful consideration of implant sizing in surgical planning. It is common practice to perform rasping to prepare the space for stem insertion. In this context, we consider that the ideal stem size would be the largest as well as the most easily insertable within the space created by gentle rasping. Creating the space that directly contacts the cortical bone solely through rasping may increase the risk of intraoperative fractures, and thereby necessitate caution. Furthermore, selecting the stem size based on this concept allows for the adequate interdigitation of cement into the remaining cancellous bone, facilitating the formation of a stable cancellous bone–cement composite, which may contribute to the prevention of postoperative fractures.

The VLIAN stem used in this study features a gradually sloping shoulder design, making it suitable for size selection even in smaller Asian femurs. While it has a smaller offset, it is particularly useful for appropriate size selection in stovepipe-type femurs, making it notably advantageous for femoral neck fractures. The shape of the stem closely resembles the shoulder design of the clinically successful Exeter stems No. 3 and No. 4, suggesting the potential for favorable long-term outcomes. In this fracture experiment, we used VLIAN’s original rasp, maintaining a cement mantle of 1 mm. The stem sizes used in this study’s experiments were appropriate for the bone, and allowed for the insertion of larger sizes compared to the Exeter stem, which requires securing a cement mantle of at least 2 mm.

Ginsel et al. reported that upsizing in Exeter stems was advantageous against fracture torque [8]. While Kaneuji et al.’s study examined relatively small No. 1 Exeter stems, the usage of larger VLIAN stems may have masked mechanical property differences due to material variations, introducing an important consideration for future research design [11]. The biomechanical interactions may have also reduced the relative impacts of material properties. This interpretation gains particular strength when considered alongside Takahashi et al.’s findings [20], which demonstrate a strong positive correlation between cement thickness and stem subsidence, showing that excessive cement layers impede effective compressive force generation. Their work revealed that smaller stems specifically tend to subside together with cement, creating an environment where material-dependent mechanical properties become more pronounced [20]. Conversely, larger stems like those used in our study may achieve more appropriate cement thickness, potentially reducing the relative impacts of material differences through optimized mechanical coupling.

This study has several important limitations that warrant careful consideration when interpreting the results. First, the relatively small sample size potentially limits both the statistical power and generalizability of our findings to broader populations. Second, as this is fundamentally a basic research study, while similar results may be anticipated in THA patients, they have not been clinically validated, necessitating further clinical verification studies. Additionally, we have not observed any PPFs in VLIAN stems at our institution, making it impossible to provide clinical reports at this time. We specifically excluded the influence of soft tissue surroundings, and in vivo conditions may significantly differ, as multiple research groups could affect both the direction of forces countering varus–torsional forces and resulting fracture patterns. Additionally, our study only demonstrated destruction torque under compression–torsion conditions, without investigating other potential failure mechanisms, such as pure torsion, bending, or direct impact forces. Third, the sizes of the bone models used in this study were limited, thus necessitating further investigations with smaller stems in small bones. Fourth, the cancellous bone in our model lacks the characteristic trabecular mesh structure, meaning the composite interface between the cancellous bone and cement is not fully reproduced. This results in a thinner cement mantle compared to cases where appropriately sized stems are inserted into actual femurs. However, this issue might not significantly impact our torque results, as all tests were conducted under identical experimental conditions. Fifth, while our experimental design reproduced bonded stem conditions, we cannot completely rule out the possibility that this bonding might have masked potential significant differences between materials. Nevertheless, it is important to note that all stems are generally considered to be bonded, and with taper-slip designs, stem fixation under loading is typically considered adequate and reliable.

Recent research has suggested that implant positioning, patient factors, and surgical technique may play crucial roles in PPF development, indicating that material properties represent just one aspect of a complex multifactorial process. The interaction between these various factors and their relative contributions to PPF risk remain important areas for future investigation. To validate our findings and explore their clinical significance more comprehensively, future research should incorporate several key elements, such as investigations across a broader range of stem sizes, longer-term follow-up studies, and significantly larger patient cohorts. Furthermore, subsequent studies would benefit from examining the complex interactions between various factors, including material properties, cement mantle thickness, surface finish variations, and patient-specific anatomical considerations. Additional research might also focus on investigating the biomechanical behavior under different loading conditions and the influence of soft tissue constraints, which could provide more comprehensive insights into the clinical performance of these implants. In the future, if the validity of FEM (Finite Element Method) analysis is proven, it will be possible to predict fracture risk under various conditions, leading to further advancements in this field.

## Figures and Tables

**Table 1:** Values of failure torque in each sample and standard deviation (N = 8) comparing Co–Cr–Mo and SUS stems. Co–Cr–Mo—cobalt–chromium–molybdenum, SUS—stainless use steel.

Samples	Failure Torque (N·m)									
	1	2	3	4	5	6	7	8	Average	Standard Deviation
**Co-Cr-Mo Alloy**	130.7	111.4	97.6	99.4	89.2	108.1	105.9	81.8	103.0	14.9
**SUS**	92.2	107.7	104.0	96.2	84.3	89.7	85.5	130.0	98.7	15.1

## Data Availability

The original contributions presented in this study are included in the article. Further inquiries can be directed to the corresponding authors.

## References

[1] Parker M.J., Cawley S. (2020). Cemented or uncemented hemiarthroplasty for displaced intracapsular fractures of the hip: A randomized trial of 400 patients. Bone Joint J..

[2] Fujita H., Katayama N., Iwase T., Otsuka H. (2012). Multi-center study of use of the Exeter stem in Japan: Evaluation of 1000 primary THA. J. Orthop. Sci..

[3] Thien T.M., Chatziagorou G., Garellick G., Furnes O., Havelin L.I., Mäkelä K., Overgaard S., Pedersen A., Eskelinen A., Pulkkinen P. (2014). Periprosthetic femoral fracture within two years after total hip replacement: Analysis of 437,629 operations in the nordic arthroplasty register association database. J. Bone Joint Surg. Am..

[4] Troelsen A., Malchau E., Sillesen N., Malchau H. (2013). A review of current fixation use and registry outcomes in total hip arthroplasty: The uncemented paradox. Clin. Orthop. Relat. Res..

[5] Palan J., Smith M.C., Gregg P., Mellon S., Kulkami A. (2016). The influence of cemented femoral stem choice on the incidence of revision for periprosthetic fracture after primary total hip arthroplasty: An analysis of national joint registry data. Bone Joint J..

[6] Scott T., Salvatore A., Woo P., Lee Y.Y., Salvati E.A., Della Valle A.G. (2018). Polished, collarless, tapered, cemented stems for primary hip arthroplasty may exhibit high rate of periprosthetic fracture at short-term follow-up. J. Arthroplast..

[7] Grammatopoulos G., Pandit H., Kambouroglou G., Deakin M., Gundle R., McLardy-Smith P., Taylor A., Murray D. (2011). A unique peri-prosthetic fracture pattern in well fixed femoral stems with polished, tapered, collarless design of total hip replacement. Injury.

[8] Ginsel B.L., Morishima T., Wilson L.J., Whitehouse S.L., Crawford R.W. (2015). Can larger-bodied cemented femoral components reduce periprosthetic fractures? A biomechanical study. Arch. Orthop. Trauma. Surg..

[9] Hirata M., Oe K., Kaneuji A., Uozu R., Shintani K., Saito T. (2021). Relationship between the Surface Roughness of Material and Bone Cement: An Increased "Polished" Stem May Result in the Excessive Taper-Slip. Materials.

[10] Goshi A., Takeda Y., Nakai T., Fukunishi S. (2024). Mechanical studies for the rotational stability of a cemented stem in cases with stem anteversion adjustment in the cement mantle. J. Orthop. Sci..

[11] Kaneuji A., Chen N., Takahashi E., Takano N., Fukui M., Soma D., Tachi I., Orita Y., Ichiseki T., Kawahara N. (2023). Collarless Polished Tapered Stems of Identical Shape Provide Differing Outcomes for Stainless Steel and Cobalt Chrome: A Biomechanical Study. J. Funct. Biomater..

[12] Huiskes R., Verdonschot N., Nivbrant B. (1998). Migration, stem shape, and surface finish in cemented hip arthroplasty. Clin. Orthop. Relat. Res..

[13] Okazaki Y. (2012). Comparison of Fatigue Properties and Fatigue Crack Growth Rates of Various Implantable Metals. Materials.

[14] Brady O.H., Garbuz D.S., Masri B.A., Duncan C.P. (2000). The reliability and validity of the Vancouver classification of femoral fractures after hip replacement. J. Arthroplast..

[15] Duncan C.P., Masri B.A. (1995). Fractures of the femur after hip replacement. Instr. Course Lect..

[16] Morishima T., Ginsel B.L., Choy G.G., Wilson L.J., Whitehouse S.L., Crawford R.W. (2014). Periprosthetic fracture torque for short versus standard cemented hip stems: An experimental in vitro study. J. Arthroplast..

[17] Müller K., Zeynalova S., Fakler J.K.M., Kleber C., Roth A., Osterhoff G. (2025). Risk factors for mortality in periprosthetic femur fractures about the hip-a retrospective analysis. Int. Orthop..

[18] Takegami Y., Osawa Y., Iida H., Okamoto M., Ozawa Y., Funahashi H., Ido H., Asamoto T., Imagama S. (2023). Addressing osteoporosis treatment after hemiarthroplasty for a femoral neck fracture: Impact on survival rates after a subsequent periprosthetic femoral fracture—A multicenter (TRON group) retrospective study. Arch. Osteoporos..

[19] Haider M.A., Garry C., Rajahraman V., Chau I., Schwarzkopf R., Davidovitch R.I., Macaulay W. (2024). Perioperative and short-term outcomes of cemented versus cementless total hip arthroplasty: A retrospective propensity-matched analysis. Arch. Orthop. Trauma. Surg..

[20] Takahashi E., Kaneuji A., Tsuda R., Numata Y., Ichiseki T., Fukui K., Kawahara N. (2017). The influence of cement thickness on stem subsidence and cement creep in a collarless polished tapered stem When Are Thick Cement Mantles Detrimental ?. Bone Joint Res..

